# A federated graph neural network framework for privacy-preserving personalization

**DOI:** 10.1038/s41467-022-30714-9

**Published:** 2022-06-02

**Authors:** Chuhan Wu, Fangzhao Wu, Lingjuan Lyu, Tao Qi, Yongfeng Huang, Xing Xie

**Affiliations:** 1 Department of Electronic Engineering, Tsinghua University, 100084 Beijing, China; 2 Microsoft Research Asia, 100080 Beijing, China; 3 Sony AI, 1-7-1 Konan Minato-ku, Tokyo, 108-0075 Japan

**Keywords:** Engineering, Business, Ethics, Machine learning

## Abstract

Graph neural network (GNN) is effective in modeling high-order interactions and has been widely used in various personalized applications such as recommendation. However, mainstream personalization methods rely on centralized GNN learning on global graphs, which have considerable privacy risks due to the privacy-sensitive nature of user data. Here, we present a federated GNN framework named FedPerGNN for both effective and privacy-preserving personalization. Through a privacy-preserving model update method, we can collaboratively train GNN models based on decentralized graphs inferred from local data. To further exploit graph information beyond local interactions, we introduce a privacy-preserving graph expansion protocol to incorporate high-order information under privacy protection. Experimental results on six datasets for personalization in different scenarios show that FedPerGNN achieves 4.0% ~ 9.6% lower errors than the state-of-the-art federated personalization methods under good privacy protection. FedPerGNN provides a promising direction to mining decentralized graph data in a privacy-preserving manner for responsible and intelligent personalization.

## Abstract (web summary)

Mainstream personalization methods rely on centralized Graph Neural Network learning on global graphs, which have considerable privacy risks due to the privacy-sensitive nature of user data. Here, the authors present a federated GNN framework for both effective and privacy-preserving personalization.

## Introduction

Personalization is a critical direction in the development of the Web^1^. It can ease the burden of information overload by providing different users with different services based on their preferences and characteristics to better satisfy their personal needs^2^. For example, personalized recommender systems can help display the products, videos and news we would like to consume^3^. Personalized healthcare services can help people’s health management and provide effective therapy plans based on an individual’s mental and physical conditions^4,5^. These personalized services have greatly empowered people in terms of informed decision making and effective interaction with the physical world^6,7^.

Advanced machine intelligence systems have played central roles in various personalized online applications such as recommendation^8^ and personalized search^9^. Due to the social nature of the Web, there are numerous interactions between users and real-world or virtual items as well as complex connections among different users^10^. Taking personalized recommendation as an example, the interactions between users and items can naturally form a bipartite graph. Mining useful information on this graph is important for understanding users and items for better personalization^11^.

Graph neural network (GNN) is an effective neural architecture for mining graph-structured data, since it can capture the high-order content and topological information on graphs^12^. It has been widely used in personalization scenarios such as product recommendation^13–15^ and content recommendation^16^ to model the complicated interactions among users and items. The success of existing GNN-based personalization systems depends on centralized graph data for model learning, which is usually constructed by the data collected from a large number of users^17^. However, user data is usually highly privacy-sensitive and its centralized storage and exploitation can lead to users’ privacy concerns and the risk of data leakage^18^. Moreover, under the pressure of some strict data protection regulations such as General Data Protection Regulation (GDPR)^19^, online platforms may not be able to centrally store user data to learn GNN models for personalization in the future^20^.

An intuitive way to tackle the privacy issue of these systems is storing raw data locally on user devices and learning local GNN models based on it. However, for most cases the data volume on user devices is too small to locally train accurate GNN models. Federated learning is a privacy-preserving machine learning paradigm that can collaboratively learn intelligent models from data decentralized on a large number of user clients under privacy protection^21^. In federated learning, only the model updates computed on the local data of clients are exchanged and aggregated, where the raw data does not leave the local devices. This paradigm enables the clients to learn their local GNN models based on the local graphs inferred from the local interaction data, and aggregates these local models into a global one for personalization, which is called subgraph-level federated learning^22^. However, two challenges still remain in this framework. First, the local GNN model trained on local user data may convey private information, and it is challenging to protect user privacy when synthesizing the global GNN model from the local ones. Second, the local user data may only contain first-order interactions between user and items, while higher-order interaction information is not available since user data cannot be directly exchanged and linked among different clients due to privacy restrictions. Prior work on subgraph-level federated learning^22^ assumes that each client has a large subgraph and there is no sufficient interaction across different subgraphs decentralized on different clients. However, in personalization scenarios the decentralized subgraphs can be very small, and the interactions across different subgraphs can be critical for understanding user interest. Thus, it is still a rather difficult problem to exploit high-order interactions to enhance GNN model learning in personalization scenarios without violating privacy protection.

In this work, we present a federated GNN framework named FedPerGNN, which can empower privacy-preserving personalization by mining high-order user-item interaction information in a privacy-preserving way. Since there is no global user-item graph due to privacy restrictions, each client needs to locally learn a GNN model based on the user-item graph constructed from the local interaction data on this device. The clients further send the model gradients to a central server, which aggregates the gradients from a number of clients and distributes the global parameter to user devices for local update. In this framework, since the model gradients contain private information, we develop a privacy-preserving model update method to protect user-item interaction information with local differential privacy (LDP) and a pseudo interacted item sampling method. To break the dilemma of information isolation, we design a privacy-preserving graph expansion protocol to exploit high-order graph information without leaking user privacy. We conduct experiments on six widely used datasets for personalization in different scenarios. The results show that FedPerGNN achieves 4.0–9.6% lower errors than several state-of-the-art (SOTA) privacy-preserving personalization methods under satisfactory privacy budget. In addition, FedPerGNN has the advantage of low communication cost and more comprehensive privacy protection than other federated personalization methods, making it a feasible choice for deployment in practice. Through extensive analysis, we also find that the information within three orders is more important for personalization, which has a certain significance for reference in designing effective, efficient and privacy-preserving personalized online systems. Our work is expected to serve as a basis workbench for future researches on privacy-preserving personalization and decentralized graph data mining.

## Result

### Overall framework

**Fig. 1:**
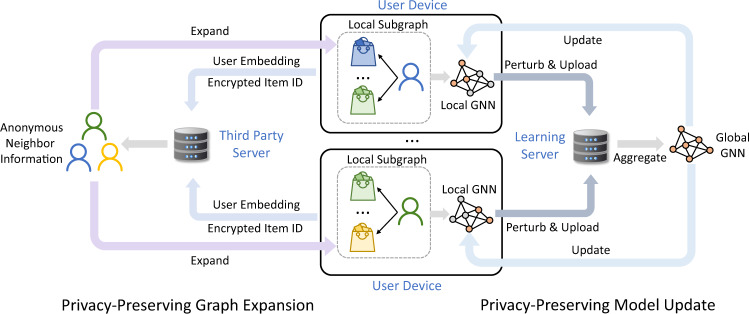
The overall framework of FedPerGNN. Each user device learns a local GNN model based on the local subgraph inferred from the local user data. A learning server is used to coordinate a large number of user devices for learning the global GNN model collaboratively. A privacy-preserving model update method is used to protect private user information encoded in the model gradients exchanged among the learning server and clients. A third-party server is used to conduct the privacy-preserving graph expansion protocol to incorporate high-order graph information into local model learning under privacy protection. The devices upload the user embedding and encrypted item IDs to this server for finding user neighbors, and the embeddings of anonymous neighbor users are distributed to user devices for expanding local subgraphs.

We first briefly introduce the overall framework of FedPerGNN for learning GNN-based personalization model in a privacy-preserving way (Fig. 1). It can leverage the highly decentralized user interaction data to learn GNN models for personalization by exploiting the high-order user-item interactions under privacy protection. The participants of FedPerGNN include a learning server to coordinate model learning, a third-party server to find and distribute anonymous neighbor information, and a large number of user clients to collaboratively learn GNN models. The user client keeps a local subgraph that consists of the user interaction histories with items and the neighbors of this user with co-interacted items with this user. The neighbor information is provided by a periodically executed privacy-preserving graph expansion process that incorporates a trusted third-party server to match encrypted items and distribute anonymous user embeddings. Each client learns the GNN models from its local subgraph, and uploads the perturbed gradients to a central learning server. The learning server is responsible for coordinating these user clients in the model learning process by aggregating the gradients received from a number of user clients and delivering the aggregated gradients to them. This process is conducted for multiple iterations until the model converges. Finally the user embeddings on the user devices are uploaded to the learning server for providing personalization services. In this way, the high-order information decentralized on different clients can be exploited to alleviate the information isolation problem, and user privacy can be well-protected.

### Performance evaluation

In our experiments, we use six widely used benchmark datasets for personalization in different scenarios. Three of them are different versions of MovieLens^23^ (with 100K, 1M, and 10M sample sizes), which are denoted as ML-100K, ML-1M and ML-10M, respectively. The others are Flixster, Douban, and YahooMusic datasets provided by^24^, and we denote YahooMusic as Yahoo. We list the detailed statistics of these datasets (Supplementary Table 1). The task on these datasets is predicting the unobserved item ratings given by users for providing future personalization.

We compare the performance of our FedPerGNN approach with several personalization methods based on the centralized storage of user data, including: probability matrix factorization (PMF)^25^, a variant of singular value decomposition (SVD++)^26^, a collaborative filtering approach with graph information named GRALS^27^, a matrix completion method with recurrent multi-graph neural networks called sRGCNN^24^, a matrix completion method named GC-MC based on graph convolutional autoencoders^28^, a graph convolution based method named PinSage^13^, neural graph collaborative filter (NGCF)^14^, and graph attention network (GAT)^29^. We also compare several SOTA privacy-preserving methods based on federated learning, including federated collaborative filtering (FCF)^30^ and FedMF^31^. We evaluate the rating prediction performance of different methods with the root mean square error (RMSE) between predicted and real ratings, and report the average results in five independent experiments with standard deviations (Table 1). We observe that the methods with high-order information on the user-item graph (e.g., GC-MC, PinSage, and NGCF) achieve better performance than those based on first-order information only (PMF). This is because modeling the high-order interactions between users and items can enhance user and item representation learning, and thereby improve the accuracy of personalization. In addition, compared with the methods based on the centralized user data storage such as GC-MC and NGCF, our approach FedPerGNN can achieve comparable or even better performance. For example, the performance difference between FedPerGNN and the best-performed baseline on the Yahoo dataset is not statistically significant (*p* > 0.1). It shows that FedPerGNN can protect user privacy and meanwhile achieve satisfactory personalization performance. Moreover, among the compared privacy-preserving personalization methods, FedPerGNN achieves the best performance. For example, compared with FedMF, the prediction error of FedPerGNN is reduced by 4.0–9.6% across different datasets (the improvement over FCF is larger), which is a significant difference (*p* < 0.001). This is because FedPerGNN can exploit high-order information of the user-item graphs in a privacy-preserving way to enhance user and item understanding. These results verify the effectiveness of FedPerGNN in privacy-preserving personalization.

**Table 1:** Performance of different methods in terms of RMSE.

Methods	Flixster	Douban	Yahoo	ML-100K	ML-1M	ML-10M
PMF	1.370 ± 0.011	0.893 ± 0.002	26.7 ± 0.529	0.970 ± 0.005	0.885 ± 0.007	0.855 ± 0.0006
SVD++	1.150 ± 0.008	0.865 ± 0.002	24.8 ± 0.498	0.948 ± 0.004	0.866 ± 0.004	0.833 ± 0.0004
GRALS	1.296 ± 0.009	0.840 ± 0.002	37.9 ± 0.786	0.933 ± 0.002	0.846 ± 0.005	0.811 ± 0.0002
sRGCNN	1.170 ± 0.007	0.805 ± 0.002	22.8 ± 0.482	0.921 ± 0.003	0.839 ± 0.003	0.785 ± 0.0003
GC-MC	0.943 ± 0.006	0.736 ± 0.001	20.4 ± 0.361	0.906 ± 0.001	0.830 ± 0.001	0.778 ± 0.0001
PinSage	0.945 ± 0.005	0.732 ± 0.001	21.0 ± 0.332	0.914 ± 0.002	0.840 ± 0.002	0.790 ± 0.0002
NGCF	0.954 ± 0.004	0.742 ± 0.001	20.9 ± 0.370	0.916 ± 0.002	0.833 ± 0.002	0.779 ± 0.0003
GAT	0.952 ± 0.005	0.737 ± 0.001	21.2 ± 0.334	0.913 ± 0.001	0.835 ± 0.001	0.784 ± 0.0004
FCF	1.064 ± 0.008	0.823 ± 0.002	22.9 ± 0.389	0.957 ± 0.002	0.874 ± 0.005	0.847 ± 0.0007
FedMF	1.059 ± 0.006	0.817 ± 0.002	22.2 ± 0.349	0.948 ± 0.002	0.872 ± 0.004	0.841 ± 0.0005
FedPerGNN	0.980 ± 0.006	0.775 ± 0.001	20.7 ± 0.325	0.910 ± 0.001	0.839 ± 0.003	0.793 ± 0.0002

Results of FedPerGNN and the best-performed baseline are in bold. The advantage of FedPerGNN over other SOTA privacy-preserving personalization methods FCF and FedMF is significant (*p* < 0.1). FedPerGNN also achieves comparable performance with other centralized GNN-based personalization methods, and there is no significant difference between FedPerGNN -based personalization methods, and there is no significant difference between FedPerGNN and the best-performed method on Yahoo (*p* > 0.1).

To show the advantage of our approach, we compare it with baseline methods in terms of exploiting high-order contexts and privacy protection (Table 2). Many existing methods rely on centralized user data storage and cannot protect user privacy. Among privacy-preserving personalization methods, they cannot exploit high-order graph information. In addition, they can only protect the private user ratings given by users, while cannot protect users’ interaction histories with items unless they store the entire set of items with their embeddings in each client, which is impractical in real-world systems. FedPerGNN can protect both ratings and user-item interaction histories, which can achieve more comprehensive privacy preservation.

**Table 2:** Comparison of different methods in high-order user-item interaction modeling and privacy protection.

	PMF	SVD++	GRALS	sRGCNN	GC-MC	PinSage	NGCF	GAT	FCF	FedMF	FedPerGNN
High-order information	×	✓	✓	✓	✓	✓	✓	✓	×	×	✓
Rating protection	×	×	×	×	×	×	×	×	✓	✓	✓
Interaction item protection	×	×	×	×	×	×	×	×	×	×	✓
User data storage	Central	Central	Central	Central	Central	Central	Central	Central	Local	Local	Local

“Central” and “Local” represent centralized and decentralized data storage, respectively. Existing centralized graph learning methods can exploit high-order graph information but cannot protect user privacy. Existing federated learning based methods can only protect private ratings given by users and they are not able to mine high-order contexts. FedPerGNN can incorporate high-order information into graph mining and meanwhile protect both user ratings and historical interaction items.

### Model effectiveness

**Fig. 2:**
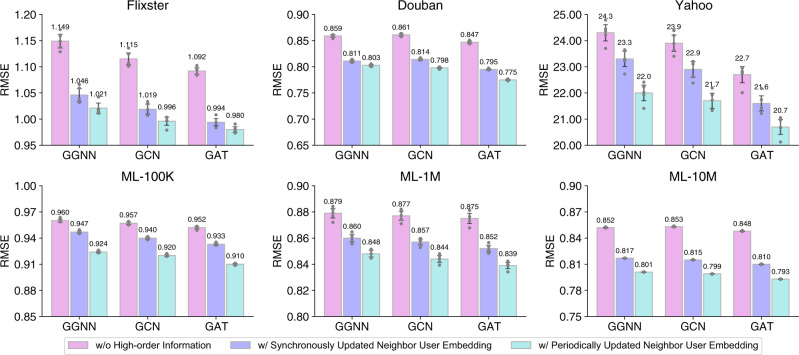
Influence of neighbor user information and different GNN architectures. The error bars represent mean results with 95% confidence intervals (*n* = 5 independent experiments). GGNN gated graph neural network, GCN graph convolution network, GAT graph attention network. The results show that incorporating the high-order information encoded in neighbor user embeddings can effectively reduce prediction errors, and using periodically updated neighbor user embedding is better than using synchronously updated ones.

Next, we validate the effectiveness of incorporating high-order information of the user-item graphs as well as the generality of our approach. We compare the performance of FedPerGNN and its variants with synchronously updated neighbor user embeddings or without high-order user-item interactions. In addition, we also compare their results under different implementations of their GNN models, including gated graph neural network (GGNN)^32^, graph convolution network (GCN)^33^ and GAT^29^. From the results (Fig. 2) we have the following findings. Compared with the baseline performance reported in Table 1, the performance of FedPerGNN and its variants implemented with other different GNN models is also satisfactory. This result shows that our approach is compatible with different GNN architectures, and thereby can serve as a general framework for GNN-based personalization. We also observe that GAT-based FedPerGNN slightly outperforms its variants based on GCN and GGNN. This is because the GAT network can more effectively model the importance of the interactions between nodes than GCN and GGNN, which is beneficial for user and item modeling. In addition, the variants that can utilize the high-order information encoded in the neighbor user embeddings perform better than those without high-order information. It validates the effectiveness of our approach in incorporating high-order information of the user-item graph into personalization. Besides, we find that using periodically updated neighbor user embeddings is slightly better than using fully trainable ones that are synchronously updated in each iteration. This may be because the neighboring user embeddings may not be accurate at the beginning of model training, and updating them synchronously is not beneficial for learning accurate user and item representations.

**Fig. 3:**
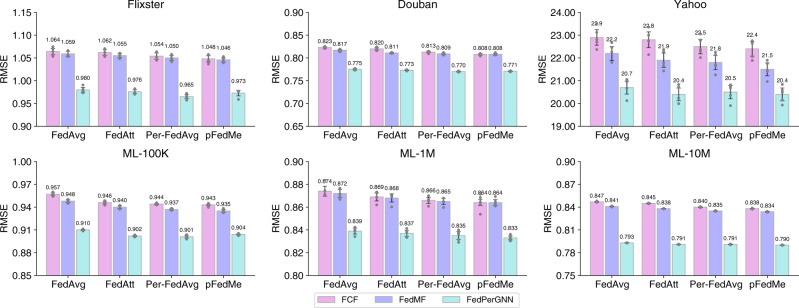
Influence of using different federated update methods on model performance. The error bars represent mean results with 95% confidence intervals (*n* = 5 independent experiments). The results show the effectiveness of FedPerGNN under different types of federated model update methods, and can slightly benefit from using more sophisticated ones.

We further analyze the effectiveness of FedPerGNN under different types of federated model update methods (Fig. 3), including FedAvg^21^, FedAtt^34^, Per-FedAvg^35^, and pFedME^36^. We compare FedPerGNN with FCF and FedMF for reference. We find that advanced federated model update methods such as FedAtt, Per-FedAvg, and pFedME slightly outperform the vanilla FedAvg method, and the personalized federated learning method Per-FedAvg and pFedME usually achieve the best performance. This is because personalized federated learning can better handle the heterogeneity of user data in personalization scenarios. In addition, we find that FedPerGNN has consistent performance improvement over other compared federated personalization methods (i.e., FCF and FedMF) under different federated model update methods. It verifies the generality of FedPerGNN in terms of being empowered by different federated learning frameworks.

### Hyperparameter analysis

**Fig. 4:**
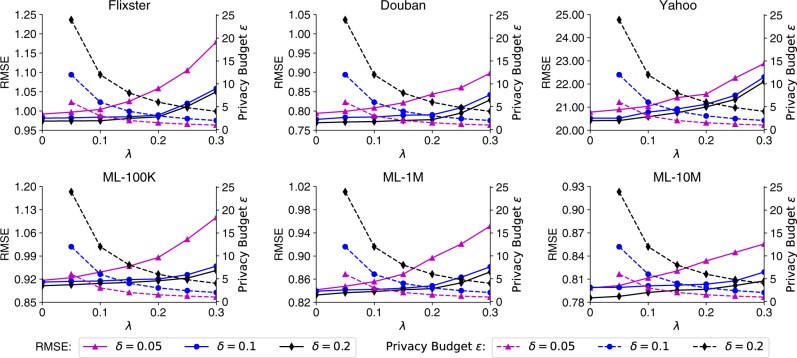
The personalization RMSE (left *y*-axis) and privacy budget *ϵ* (right *y*-axis) w.r.t. different clipping threshold *δ* and noise strength *λ*. Lower RMSE means better performance and lower privacy budget means better privacy protection. The privacy budget under *λ* = 0 is infinite. The performance sacrifice is similar when the clipping threshold *δ* is 0.1 or 0.2, while it becomes much heavier when *δ* is 0.05. The performance loss is also larger when a stronger noise strength is used. FedPerGNN achieves 3-differential privacy under *δ* = 0.1 and *λ* = 0.2 (the number of epochs is 3).

We then study the influence of several important hyperparameters on different aspects of FedPerGNN, including performance, privacy protection, and communication cost. We first compare the performance and privacy budget of our FedPerGNN approach by varying the gradient clipping threshold *δ* and the strength *λ* of Laplacian noise in the LDP module (Fig. 4). A larger *λ* and smaller *δ* means a smaller budget *ϵ*, i.e., better privacy protection. According to these results, we find model performance gap between *δ* = 0.1 and *δ* = 0.2 is marginal. However, if we clip the gradients with a smaller threshold such as 0.05, the prediction error will substantially increase. Thus, we set *δ* = 0.1 due to the better privacy protection without much sacrifice of model performance. In addition, the model performance declines with the growth of the noise strength *λ*, while the performance loss is not too heavy if *λ* is moderate. Thus, we set *λ* to 0.2 to achieve a good balance between privacy protection (we achieve 3-differential privacy under this setting) and personalization accuracy.

**Fig. 5:**
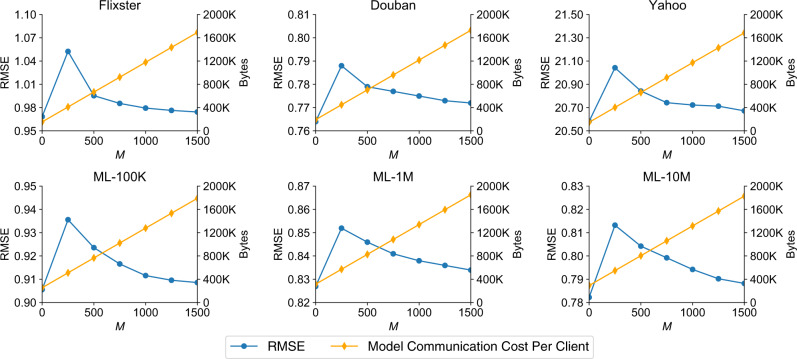
The personalization RMSE (left *y*-axis) and communication cost (right *y*-axis) under different numbers of pseudo interacted items (*M*). The communication cost is the same for both downloading and uploading. The performance is optimal when there is no pseudo interacted item, but users' interaction histories are not protected. When the value of *M* is too small the performance loss is relatively large, while the performance improves and user privacy is better protected when *M* becomes larger. Since the communication cost is proportional to *M*, a moderated value of *M* (i.e., 1000) is chosen.

We also compare the performance and communication cost of FedPerGNN w.r.t. different *M* (Fig. 5). We use the number of parameters to be exchanged in each iteration during model training to measure the communication cost. From the results we observe an interesting peak on the performance curve. This is because the performance is the best if *M* is 0, but the user-item interaction histories cannot be protected. In addition, the performance declines when *M* is non-zero because the randomly generated gradients will affect the accuracy of item gradients, while the performance sacrifice is smaller when using a larger value of *M*. This is because when *M* is relatively large, the random gradients of pseudo interacted items can be better counteracted after aggregation and their influence is better mitigated. Moreover, the model can achieve at least $\frac{1000}{M}$-index privacy on the Movielens datasets (discussed in detail in the Methodology Section), and privacy protection on other datasets is better. Thus, if *M* is too small the user privacy cannot be well-protected. However, the communication cost is also proportional to *M* and can be heavy if *M* is too large. Since the performance improvement under *M* > 1000 is marginal, we set *M* to 1000 to achieve 1-index privacy and good personalization performance, where the communication cost is reasonable.

**Fig. 6:**
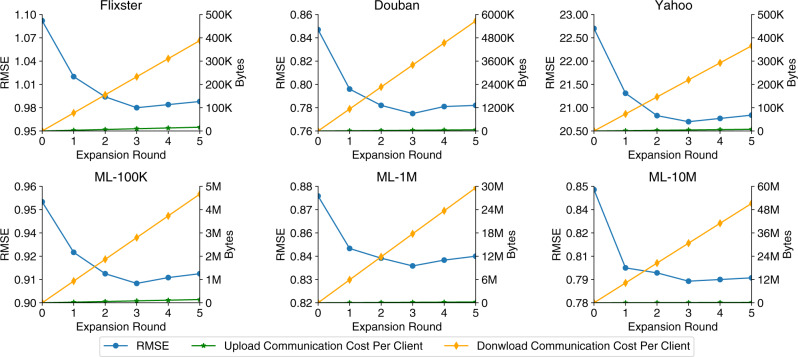
The personalization RMSE and upload/download communication cost under different numbers of graph expansion rounds. The performance is optimal when there are three expansion rounds. The first two rounds of expansion contribute most to the performance improvements, which indicate the graph information in the first three orders is more important for personalization. The communication cost is proportional to the expansion rounds, and the download communication cost is much larger than upload.

We further evaluate the performance and upload/download communication cost of FedPerGNN under different rounds of privacy-preserving graph expansion (Fig. 6). We find that the errors decrease when the expansion round increases from 0 to 3, and the improvements are mainly brought by the first two rounds of graph expansion. This phenomenon indicates that the graph information in the first three orders plays the most important role in personalization. In addition, the performance decreased when there are too many expansion rounds, which is probably because of the over-smooth problem in GNN models^37^. Besides, we find the major communication cost is brought by downloading the anonymous neighbor user embeddings, which is proportional to the expansion round. Thus, in our approach we use three rounds of expansion to achieve the best performance within an acceptable communication cost. Since the download bandwidth is usually more abundant than upload bandwidth^38^, FedPerGNN is practical in real-world scenarios.

## Discussion

In this work we present FedPerGNN, a federated framework for privacy-preserving GNN-based personalization, which aims to collaboratively train GNN models from decentralized user data by exploiting high-order interaction information in a privacy-preserving manner. In our method, we allow each user client to locally train a GNN model based on its local user-item graph stored on this device. Each client uploads the locally computed gradients to a server for aggregation, which are further sent to user clients for local updates. Since the communicated model gradients may contain private user information, we develop a privacy-preserving model update method to protect user privacy in model training. Different from existing methods that can only protect private user ratings, our method can protect both ratings and interaction histories, which can achieve more comprehensive privacy preservation in practice. In addition, our method does not need to communicate and locally memorize the global item set, and its communication overhead is usually acceptable for modern personal devices. Thus, FedPerGNN can be easier to be deployed in real-world personalization services.

Since the local user-item graphs inferred from local user data only contain low-order interaction information, we propose a privacy-preserving user-item graph expansion protocol to extend local graphs and propagate high-order information under privacy protection. In this process, each client receives the anonymous user embeddings to expand the local subgraph, which helps the propagation of high-order information on the user-item graph in a privacy-preserving manner to enhance the performance of GNN model. Within only a few rounds of privacy-preserving graph expansion, the high-order information on the user-item graph can be effectively exploited without heavy communication cost. In addition, this method is not limited to the personalization scenario and can serve as a basic technique for privacy-preserving data mining on decentralized graph data, which has the potential to facilitate researches in various fields that involve graph-structured data.

We conducted extensive experiments on six real-world datasets under different scenarios. The results show that FedPerGNN can achieve competitive performance with existing GNN methods based on centralized data storage, and can achieve 4.0–9.6% lower prediction errors than SOTA privacy-preserving methods. The experimental results further validate the generality of FedPerGNN in boosting the performance of GNN models with various architectures, which shows the potential of our method in serving as a general benchmark for privacy-preserving GNN model learning. We also find that FedPerGNN can achieve a good balance between accuracy, privacy protection and communication cost, which provides great potential to be incorporated in practice. Through the analysis of graph expansion, we find the graph information within the first three orders takes the core role in personalization, which may provide useful guidance for researchers to reveal the inherent mechanism of GNN model and help practitioners develop both effective and efficient graph modeling systems.

The FedPerGNN method we proposed can be used as a template framework for mining decentralized graph data under privacy protection. It is friendly to clients with limited communication resources, and is compatible with a large number of clients for collaborative model learning. FedPerGNN also provides the potential to empower many other scenarios that involve private graph data, such as intelligent healthcare, urban computing, and quantitative finance. We hope it can inspire future researches in other related fields to improve the effectiveness and responsibility of machine intelligence systems.

However, FedPerGNN has the following limitations. First, FedPerGNN relies on the assumption that third-party server is trusted and does not collude with the recommendation server, which is somewhat strong. Second, FedPerGNN may be brittle to attackers with a large number of malicious clients. Thus, in our future work, we will study how to defend against intended attacks from malicious clients and platforms. Furthermore, we plan to explore the effective and secure deployment of FedPerGNN in real-world personalization systems to serve their users under privacy preservation.

## Methods

In this section, we first introduce the problem definitions in our FedPerGNN framework, then introduce the details of our FedPerGNN approach, and finally provide some discussions and analysis on privacy protection.

### Problem formulation

Denote ${{{{{{{\mathcal{U}}}}}}}}=\{{u}_{1},{u}_{2},...,{u}_{P}\}$ and ${{{{{{{\mathcal{T}}}}}}}}=\{{t}_{1},{t}_{2},...,{t}_{Q}\}$ as the sets of users and items respectively, where *P* is the number of users and *Q* is the number of items. Denote the rating matrix between users and items as ${{{{{{{\bf{Y}}}}}}}}\in {{\mathbb{R}}}^{P\times Q}$, which is used to form a bipartite user-item graph ${{{{{{{\mathcal{G}}}}}}}}$ based on the observed ratings **Y**_*o*_. We assume that the user *u*_*i*_ has interactions with *K* items, which are denoted by [*t*_*i*,1_, *t*_*i*,2_, . . . , *t*_*i*,*K*_]. These items and the user *u*_*i*_ can form a first-order local user-item subgraph ${{{{{{{{\mathcal{G}}}}}}}}}_{i}$ (the non-shaded area in Supplementary Fig. 4). The ratings that given to these items by user *u*_*i*_ are denoted by [*y*_*i*,1_, *y*_*i*,2_, . . . , *y*_*i*,*K*_]. To protect user privacy (both the private ratings and the items a user has interactions with), each user device locally keeps its individual interaction data, and the raw data never leaves the user device. We aim to predict the user ratings based on the interaction data ${{{{{{{{\mathcal{G}}}}}}}}}_{i}$ locally stored on user devices in a privacy-preserving way. Note that there is no global user-item interaction graph in our approach and local graphs are built and stored in different devices, which is essentially different from existing federated GNN methods^22,39,40^ that require the entire graph to be built and stored together in at least one platform or device.

### FedPerGNN framework

**Fig. 7:**
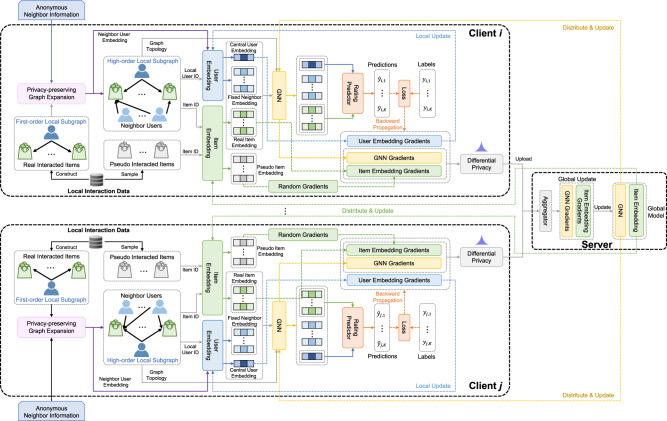
The detailed framework of FedPerGNN. Each client locally stores the interaction data and constructs the first-order local subgraph from it. This graph is expanded by the neighbor users. Several pseudo interacted items are also sampled from the local interaction data to hide real interacted items. The neighbor user embeddings are fixed, and the central user embedding is locally updated. The item embedding and GNN gradients are perturbed before uploading to a server for aggregation, and the aggregated ones are delivered to clients for local update.

Next, we introduce the details of FedPerGNN to train GNN-based personalization model in a privacy-preserving way (Fig. 7). The local subgraph on each user client is constructed from the user-item interaction data and the neighboring users that have co-interacted items with this user. The node of this user is connected to the nodes of the items it interacted with, and these item nodes are further connected to the anonymous neighboring users. An embedding layer is first used to convert the user node *u*_*i*_, the *K* item nodes [*t*_*i*,1_, *t*_*i*,2_, . . . , *t*_*i*,*K*_] and the *N* neighboring user nodes [*u*_*i*,1_, *u*_*i*,2_, . . . , *u*_*i*,*N*_] into their embeddings, which are denoted as ${{{{{{{{\bf{e}}}}}}}}}_{i}^{u}$, $[{{{{{{{{\bf{e}}}}}}}}}_{i,1}^{t},{{{{{{{{\bf{e}}}}}}}}}_{i,2}^{t},...,{{{{{{{{\bf{e}}}}}}}}}_{i,K}^{t}]$ and $[{{{{{{{{\bf{e}}}}}}}}}_{i,1}^{u},{{{{{{{{\bf{e}}}}}}}}}_{i,2}^{u},...,{{{{{{{{\bf{e}}}}}}}}}_{i,N}^{u}]$, respectively. Since the user embeddings may not be accurate enough when the model is not well-tuned, we first exclude the neighboring user embeddings at the beginning of model learning, and then incorporate them into model learning when they have been tuned. Note that the embeddings of the user *u*_*i*_ and the item embeddings are synchronously updated during model training, while the embeddings of neighboring users are periodically updated.

Next, we apply a graph neural network to these embeddings to model the interactions between nodes on the local first-order sub-graph. Various kinds of GNN networks can be used in our framework, such as GCN^33^, GGNN^32^ and GAT^29^. The GNN model outputs the hidden representations of the user and item nodes, which are denoted as ${{{{{{{{\bf{h}}}}}}}}}_{i}^{u}$, $[{{{{{{{{\bf{h}}}}}}}}}_{i,1}^{t},{{{{{{{{\bf{h}}}}}}}}}_{i,2}^{t},...,{{{{{{{{\bf{h}}}}}}}}}_{i,K}^{t}]$ and $[{{{{{{{{\bf{h}}}}}}}}}_{i,1}^{u},{{{{{{{{\bf{h}}}}}}}}}_{i,2}^{u},...,{{{{{{{{\bf{h}}}}}}}}}_{i,N}^{t}]$, respectively. Then, a rating predictor module is used to predict the ratings given by the user *u*_*i*_ to her interacted items (denoted by $[{\hat{y}}_{i,1},{\hat{y}}_{i,2},...,{\hat{y}}_{i,K}]$) based on the embeddings of items and this user. These predicted ratings are compared against the gold ratings locally stored on the user device to compute the loss function. For the user *u*_*i*_, the loss function ${{{{{{{{\mathcal{L}}}}}}}}}_{i}$ is computed as ${{{{{{{{\mathcal{L}}}}}}}}}_{i}=\frac{1}{K}\mathop{\sum }\nolimits_{j = 1}^{K}| {\hat{y}}_{i,j}-{y}_{i,j}{| }^{2}$. We use the loss ${{{{{{{{\mathcal{L}}}}}}}}}_{i}$ to derive the gradients of the models and embeddings, which are denoted by ${{{{{{{{\bf{g}}}}}}}}}_{i}^{m}$ and ${{{{{{{{\bf{g}}}}}}}}}_{i}^{e}$, respectively. These gradients will be further uploaded to the server for aggregation.

The server aims to coordinate all user devices and compute the global gradients to update the model and embedding parameters in these devices. In each round, the server awakes a certain number of user clients to compute gradients locally, which are then sent to the server. After the server receives the gradients from these users, the aggregator in this server will aggregate these local gradients into a unified one **g**. We use the FedAvg^21^ algorithm to implement the aggregator. Then, the server sends the aggregated gradients to each client to conduct local parameter update. Denote the parameter set in the *i*-th user device as Θ_*i*_. It is updated by Θ_*i*_ = Θ_*i*_ − *α***g**, where *α* is the learning rate. This process will be iteratively executed until the model converges. When the model learning process completes, the user clients will upload their locally inferred hidden user embeddings to the server for providing future personalization services. We summarize the learning framework of our FedPerGNN method (Supplementary Algorithm 1). We then introduce two modules for privacy protection in FedPerGNN, i.e., a privacy-preserving model update module (corresponding to Lines 9–13 in Algorithm 1) for protecting gradients in the model update and a privacy-preserving user-item graph expansion module (corresponding to Line 15 in Algorithm 1) to protect user privacy when modeling high-order user-item interactions.

### Privacy-preserving model update

If we directly upload the GNN model and item embedding gradients, then there may be some privacy issues due to the following reasons. First, for embedding gradients, only the items that a user has interactions with have non-zero gradients to update their embeddings, and the server can directly recover the full user-item interaction history based on the non-zero item embedding gradients. Second, besides the embedding gradients, the gradients of the GNN model and rating predictor may also leak private information of user histories and ratings^41^, because the GNN model gradients encode the preferences of users on items. In existing methods such as FedMF^31^, homomorphic encryption techniques are applied to gradients to protect private ratings. However, in this method the user device needs to locally memorize the embedding table of the entire item set ${{{{{{{\mathcal{T}}}}}}}}$ and upload it in every iteration to achieve user interaction history protection, which is impractical due to the huge storage and communication costs during model training.

To tackle these challenges, we propose two strategies to protect user privacy in the model update process. The first one is pseudo interacted item sampling. Concretely, we sample *M* items that the user has not interacted with. and randomly generate their gradients ${{{{{{{{\bf{g}}}}}}}}}_{i}^{p}$ using a Gaussian distribution with the same mean and co-variance values with the real item embedding gradients. Note that there are many sampling methods such as using the displayed items that have no interaction with a user. In our experiments we randomly sample items from the full item set for simulation. The real embedding gradients ${{{{{{{{\bf{g}}}}}}}}}_{i}^{e}$ are combined with the pseudo item embedding gradients ${{{{{{{{\bf{g}}}}}}}}}_{i}^{p}$, and the unified gradient of the model and embeddings on the *i*-th user device (Line 27 in Algorithm 1) is modified as ${{{{{{{{\bf{g}}}}}}}}}_{i}=({{{{{{{{\bf{g}}}}}}}}}_{i}^{m},{{{{{{{{\bf{g}}}}}}}}}_{i}^{e},{{{{{{{{\bf{g}}}}}}}}}_{i}^{p})$. The second one is LDP. Following^42^, we clip the local gradients on user clients based on their L1-norm with a threshold *δ*, and apply a LDP^43^ module with zero-mean Laplacian noise to the unified gradients to achieve better user privacy protection, which are formulated as follows:

1 $${{{{{{{{\bf{g}}}}}}}}}_{i}=clip({{{{{{{{\bf{g}}}}}}}}}_{i},\delta )+Laplace(0,\lambda ),$$

where *λ* is the noise scale. The privacy budget *ϵ* can be bounded by $\frac{2\delta e}{\lambda }$, where *e* is the number of epochs. The protected gradients **g**_*i*_ are uploaded to the learning server for aggregation.

### Privacy-preserving user-item graph expansion

**Fig. 8:**
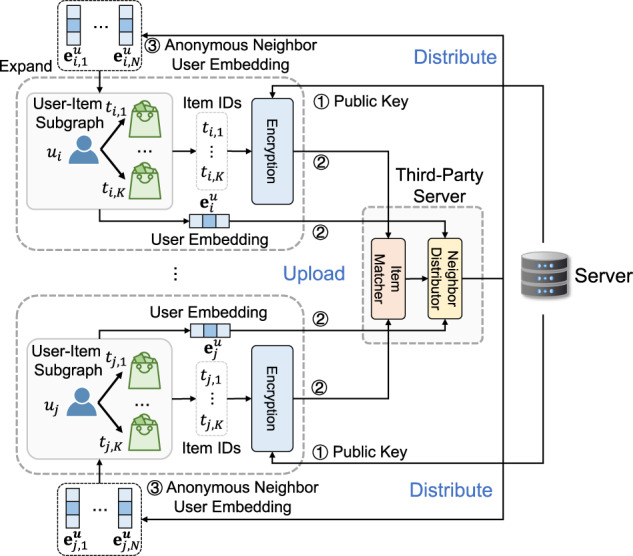
The framework of the privacy-preserving user-item graph expansion protocol. The server first generates and sends a public key to clients for encrypting local item IDs, and the clients upload the ciphertexts to a third-party server for matching the same items. The users with co-interacted items are regarded as neighbors, and the anonymous neighbor user embeddings with their corresponding connected encrypted items are distributed to the clients for expanding local subgraphs.

Then, we introduce our privacy-preserving user-item graph expansion protocol that aims to find the neighbors of users and extend the local user-item graphs in a privacy-preserving way. In existing GNN-based personalization method based on centralized graph storage, high-order user-item interactions can be directly derived from the global user-item graph. However, when user data is decentralized, it is a non-trivial task to incorporate high-order user-item interactions without violating user privacy protection. To solve this problem, we design a privacy-preserving user-item graph expansion protocol that finds the anonymous neighbors of users to enhance user and item representation learning, while protecting user privacy. Its framework is shown in Fig. 8. The central learning server that maintains the personalization services first generates a public key, and then distributes it to all user clients for encryption. After receiving the public key, each user device applies Rivest–Shamir–Adleman encryption to the IDs of the items he/she interacted with, because the IDs of these items are privacy-sensitive. The encrypted item IDs as well as the embedding of this user are uploaded to a trusted third-party server. This server finds the users who interacted with the same items by matching the ciphertexts of item IDs, and then provides each user with the embeddings of his/her anonymous neighbors. In this stage, the server for personalization never receives the private information of users, and the third-party server cannot obtain any private information of users and items since it cannot decrypt the item IDs. We connect each anonymous user node with its interacted item nodes. In this way, the local user-item subgraphs can be enriched by the high-order user-item interactions without harming the protection of user privacy. The example structure of the expanded local user-item subgraphs is shown in Supplementary Fig. 4, and the shaded area is added by the graph expansion method. We summarize the process of our privacy-preserving user-item graph expansion protocol (Supplementary Algorithm 2).

### Analysis on privacy protection

Overall, user privacy is protected from four aspects in our FedPerGNN approach. First, in FedPerGNN the personalization server never collects raw user-item interaction data, and only local computed gradients are uploaded to this server. Based on the data processing inequality, we can infer that these gradients contain much less private information than the raw user interaction data^21^. Second, the third-party server also cannot infer private information from the encrypted item IDs since it cannot obtain the private key. However, if the personalization server colludes with the third-party server by exchanging the private key and item table, the user interaction history will not be protected. Fortunately, the private ratings can still be protected by our privacy-preserving model update method. Third, in FedPerGNN we propose a pseudo interacted item sampling method to protect the real interacted items by sampling a number of items that have not been interacted by a user. Since gradients of both kinds of items have the same mean and co-variance values, it is difficult to discriminate the real interacted items from the pseudo ones if the number of pseudo interacted items is sufficiently large. It is proved in^44^ that FedPerGNN can achieve $\frac{K}{M}-$index privacy, and a smaller index privacy value indicates better privacy protection. Thus, the number of pseudo interacted items can be relatively larger to achieve better privacy protection as long as the computation resources of user devices permit. Fourth, we apply the LDP technique to the gradients locally computed by the user device, making it more difficult to recover the raw user consumption history from these gradients. Prior work^42^ has shown that the upper bound of the privacy budget *ϵ* is $\frac{2\delta }{\lambda }$, which means that we can achieve a smaller privacy budget *ϵ* by using a smaller clipping threshold *δ* or a larger noise strength *λ* to achieve better privacy protection. However, the model gradients will be inaccurate if the privacy budget is too small. Thus, we need to properly choose both hyperparameters to balance model performance and privacy protection.

### Reporting summary

Further information on research design is available in the Nature Research Reporting Summary linked to this article.

## Supplementary information

Supplementary Information

Reporting Summary

## Data Availability

The datasets involved in this study are all publicly available ones, and we adhere to the original licenses of them when conducting experiments and analysis. The MovieLens datasets (100K, 1M, and 10M versions) are available at https://grouplens.org/datasets/movielens/. The Flixster, Douban, and YahooMusic datasets are available at https://github.com/fmonti/mgcnn. Source data are provided with this paper.

## Source data

Source Data

## Code availability

Code used for this study is available at https://github.com/wuch15/FedPerGNN^45^. In addition, all experiments and implementation details are described in sufficient detail in the Methodology section and in the Supplementary Information for reproducibility.
