# Supplementary material for: A federated graph neural network framework for privacy-preserving personalization
Source: Nat Commun. 2022 Jun 2;13:3091. doi: 10.1038/s41467-022-30714-9 (PMC9163103; doi:10.1038/s41467-022-30714-9)
Supplement: Supplementary file 1 — Supplementary Information [file 41467_2022_30714_MOESM1_ESM.pdf]

Supplementary Information for "A Federated Graph Neural Network Framework for Privacy-Preserving Personalization"

Chuhan Wu[1], Fangzhao Wu[2*], Lingjuan Lyu[3], Tao Qi[1], Yongfeng Huang[1*], and Xing Xie[2]

[1]Department of Electronic Engineering, Tsinghua University, Beijing 100084, China
[2]Microsoft Research Asia, Beijing 100080, China
[3]Sony AI, 1-7-1 Konan Minato-ku, Tokyo, 108-0075 Japan
[*]Correspondence: fangzwu@microsoft.com, yfhuang@tsinghua.edu.cn

Supplementary Materials

Datasets

In our experiments, following[1] we use six widely used benchmark datasets for personalization, including MovieLens (available at https://grouplens.org/datasets/movielens/) (100K, 1M, and 10M versions), Flixster, Douban, and YahooMusic. We use the pre-processed subsets of the Flixster, Douban, and YahooMusic datasets provided by[2] (available at https://github.com/fmonti/mgcnn). We summarize the detailed statistics of these datasets (Supplementary Table 1). We also provide the distributions of the number of interacted items of a user, the number of interacted users on an item, and the number of neighbor users of a user with co-interacted items (Supplementary Figures 1, 2 and 3) To further understand the characteristic of the local subgraph on each client, we visualize the number of user/item nodes (Supplementary Figure 5) as well as their average degrees (Supplementary Figure 6). We find the number of user nodes is usually larger than the number of item nodes, and the average degrees of item nodes are usually much larger than the average degrees of user nodes. This shows that user interest is diverse, and the items interacted by a user are not consumed by a very small group of users.

Following[1], on the Flixster, Douban and Yahoo datasets we use 80/20 training/test set splits, and sample 20% of validation data from the training set when tuning hyperparameters. For the ML-100K dataset, the performance is evaluated on the given train/test split. The model hyperparameters are tuned on 20% of validation data randomly sampled from the training set. On the ML-1M or ML-10M dataset, the performance is evaluated on the same five 90/10 training/test set splits. We also sample 5% of validation data from the training set.

Experimental Settings

Hyperparameter Settings

In our experiments, we use graph attention network (GAT)[3] as the GNN model for demonstration, and use dot product to implement the rating predictor. The user and item embeddings and their hidden representations learned by graph neural networks are 256-dim. The gradient clipping threshold δ is set to 0.1, and the strength of Laplacian noise in the LDP module is set to 0.2. The number of pseudo interacted items is set to 1,000. The number of users chosen in each round of model training is 128, and the total number of epoch is 3. We update the neighbor user embeddings after every epoch, i.e., 3 rounds in total. Since on the ML-1M dataset the number of neighbor users can be too large, we limit the maximum number of neighbor users to 10,000 for efficiency consideration. The ratio of dropout[4] is 0.2. SGD is selected as the optimization algorithm, and its learning rate is 0.01. The metric used in our experiments is rooted mean square error (RMSE), and we report the average RMSE scores over 5 repetitions.

Experimental Environment

All experiments are conducted on a Linux server installed with Ubuntu 16.04 operating system and Python 3.6.9. The CPU type is Intel Xeon E5-2620 v4, and the type of GPU is GeForce GTX1080Ti with 12 GB memory. The total memory is 64GB. We use Keras 2.2.4 with the tensorflow 1.12 backend to implement our models. Analysis was performed by numpy 1.19.5, scipy 1.5.2 and pandas 1.1.5. Each experiment is run on a single GPU and CPU core with a single process/thread.

Time Cost Analysis

We analyze the time cost of the proposed method (Supplementary Figure 7) to verify its efficiency. Since many real-world edge devices do not have GPUs, we evaluate both training and inference time using the CPU mode. We find on all datasets, the local training and inference task on each sample can be done within tens of milliseconds, which is acceptable in real-world applications. The results show that FedPerGNN is feasible to be deployed in real-world federated learning systems.

Impact of Hidden Dimension

We study the influence of the hidden dimension (the embedding and GNN model dimensions) on the model performance by varying the hidden dimension from 32 to 1024 (Supplementary Figure 8). We find the performance first improves with the increase of hidden model size. This is because low-dimensional vectors may be insufficient to encode user and item characteristics. However, the performance also declines when the hidden dimension is too large. This is because the too many learnable parameters will increase the risk of overfitting. Thus, we set the hidden dimension to 256 that achieves good performance consistently on all datasets.

References

1. Berg, R. v. d., Kipf, T. N. & Welling, M. Graph convolutional matrix completion. *arXiv preprint arXiv:1706.02263* (2017).

2. Monti, F., Bronstein, M. & Bresson, X. Geometric matrix completion with recurrent multi-graph neural networks. In *NIPS*, 3697–3707 (2017).

3. Velickovic, P. *et al.* Graph attention networks. In *ICLR* (2018).

4. Srivastava, N., Hinton, G. E., Krizhevsky, A., Sutskever, I. & Salakhutdinov, R. Dropout: a simple way to prevent neural networks from overfitting. *JMLR* **15**, 1929–1958 (2014).

Supplementary Figures

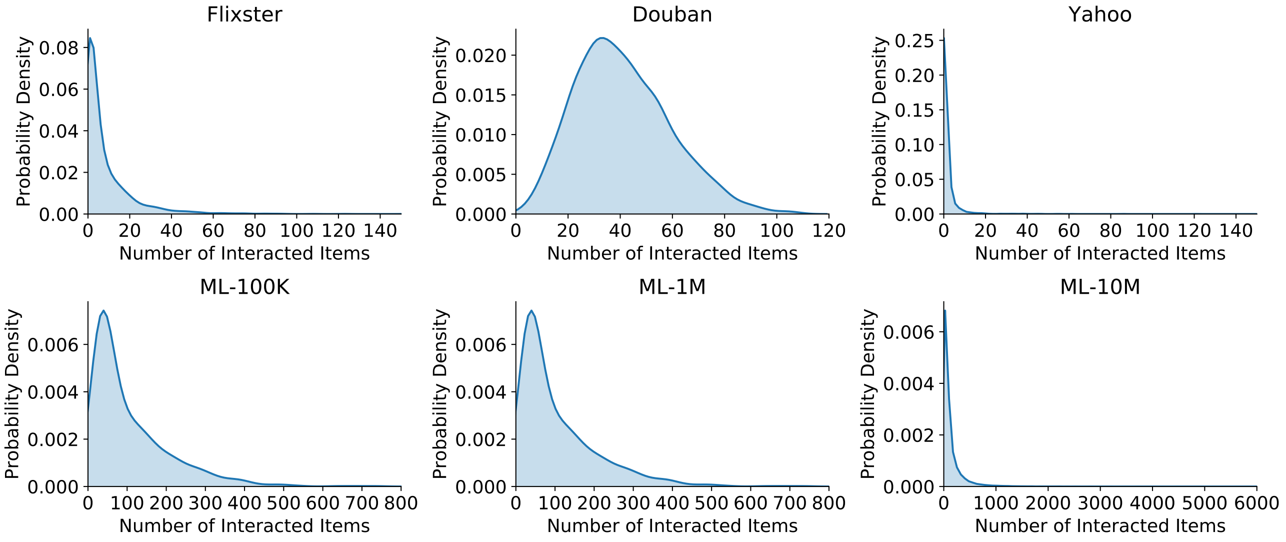

Supplementary Figure 1. Distributions of the number of historical interacted items in the user interaction history on the six datasets. Users in different datasets have very different number of interacted items, which indicate different user activeness. The distributions on all datasets are long-tail curves.

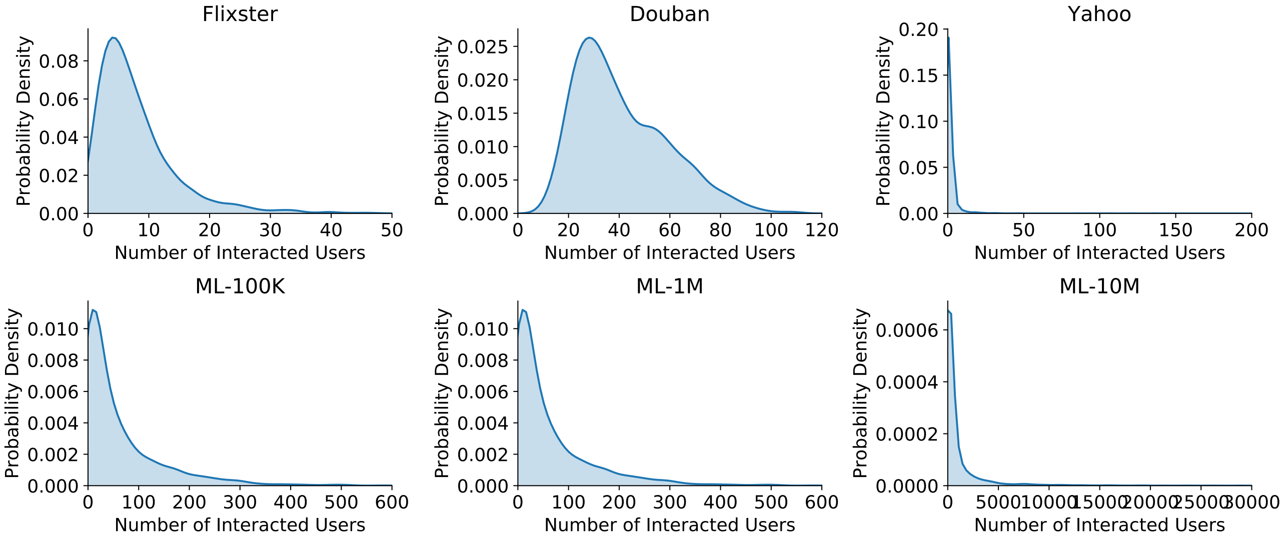

Supplementary Figure 2. Distributions of the number of users interacted with an item on the six datasets. The different numbers of interacted users indicate different popularity in the corresponding dataset. The distributions on all datasets are also long-tail curves.

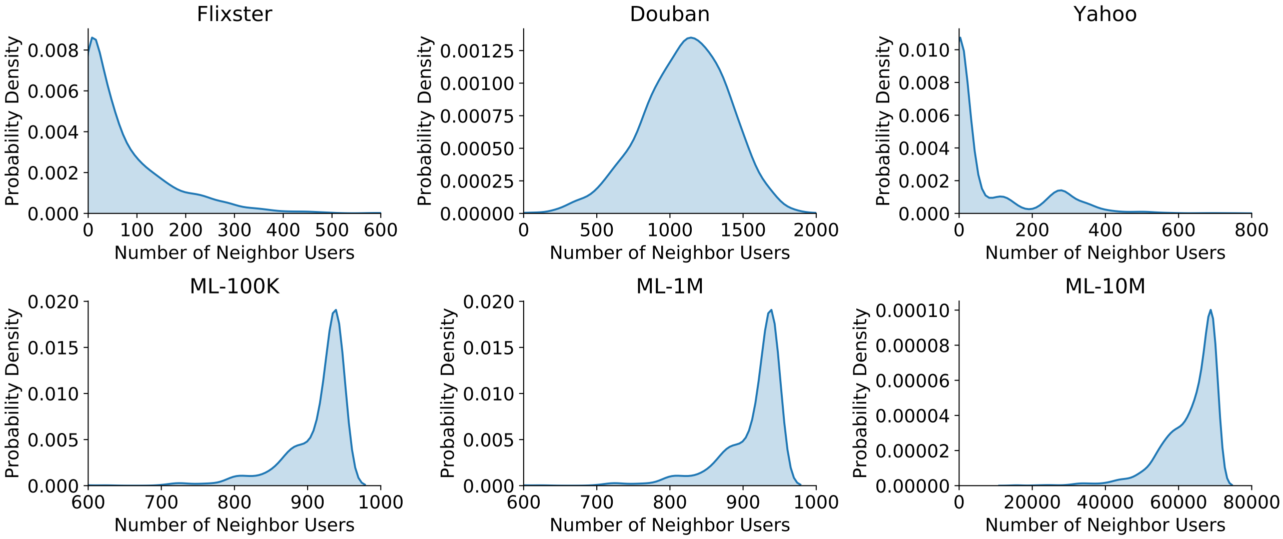

Supplementary Figure 3. The distribution of the number neighbor users with co-interacted items. Different numbers of neighbors indicate the number of users with potential related interests. We find on some datasets like Yahoo, the curve has multiple peaks, which may indicate that there are some inherent interest "clubs" in the dataset.

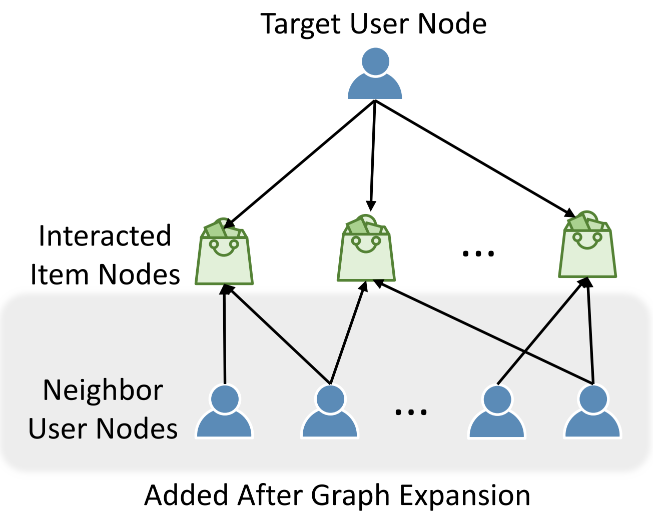

Supplementary Figure 4. An example structure of local user-item subgraphs. A subgraph on each target user device contains a node of this target user and a set of nodes that represent interacted items. After the graph expansion operation, it further integrates nodes of neighbor users that have co-interacted items with the target user.

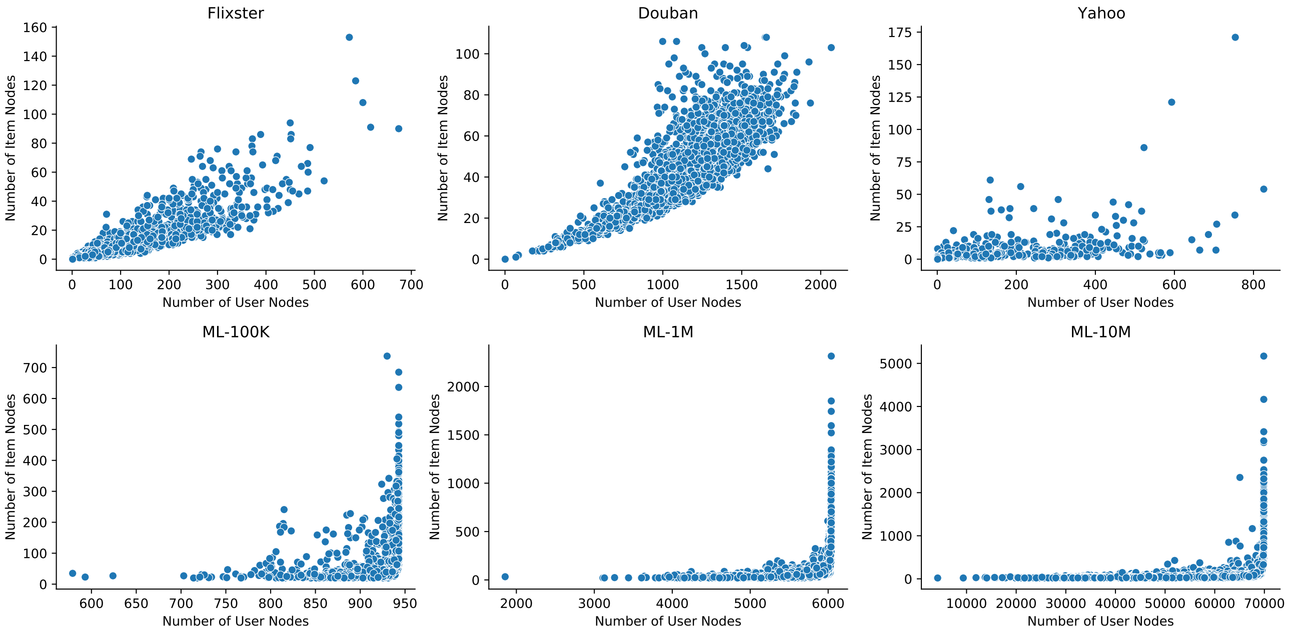

Supplementary Figure 5. The numbers of user and item nodes in each local subgraph. Most subgraphs have much more user nodes than item nodes.

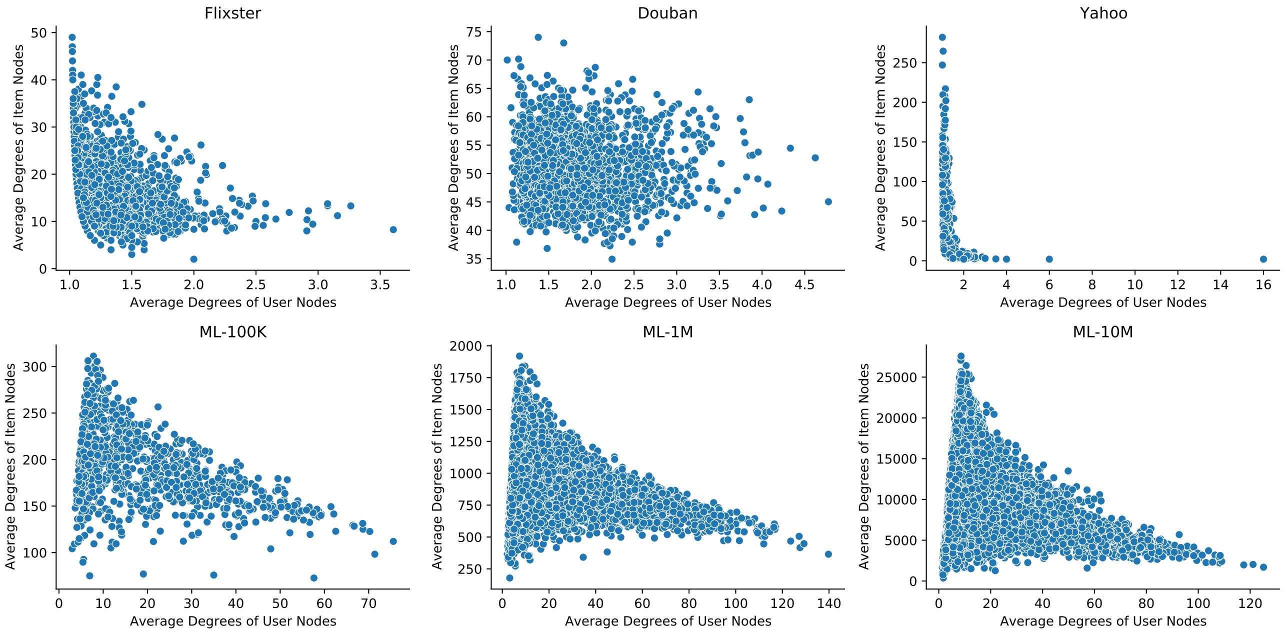

Supplementary Figure 6. The average degrees of user and item nodes in each local subgraph. The degrees of item nodes are usually much larger than those of user nodes.

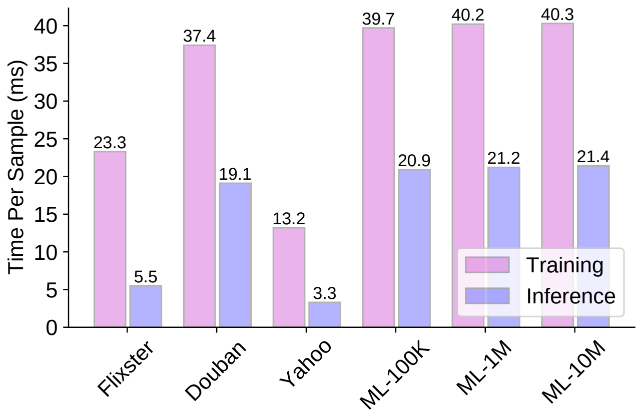

Supplementary Figure 7. The average training and inference time of each sample on different datasets. The datasets with denser user behaviors usually yield slightly higher time costs.

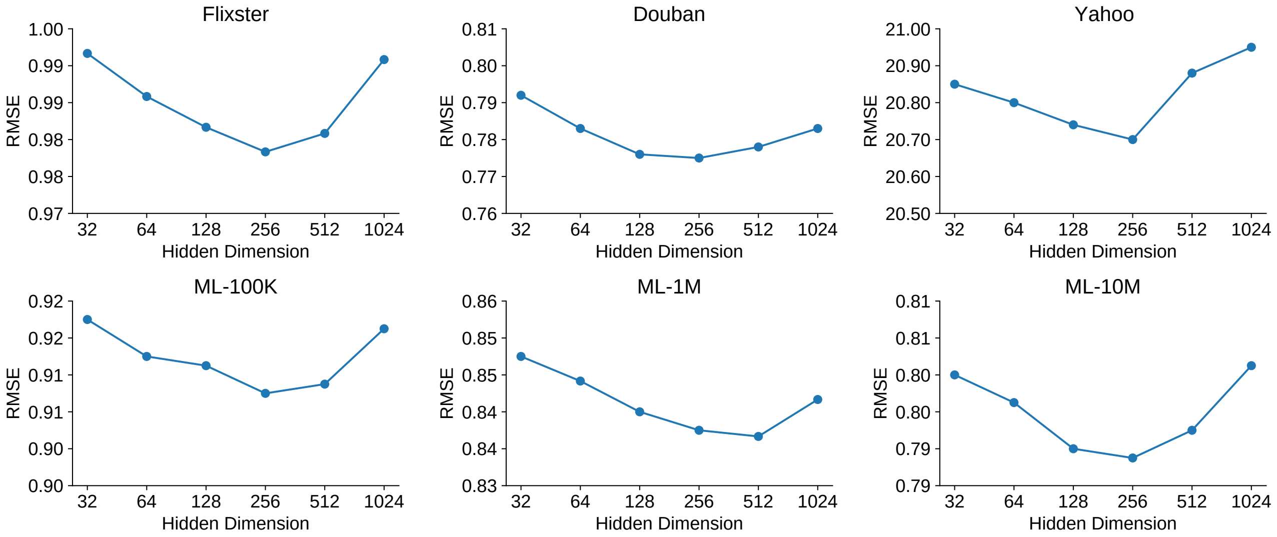

Supplementary Figure 8. Influence of the hidden model dimension on the model performance of FedPerGNN. Lower RMSE indicates better performance. The prediction errors first decrease and then raise with the increase of hidden dimension. The optimal hidden dimension in FedPerGNN is around 256.

Supplementary Tables

Dataset	#Users	#Items	#Ratings	Rating Levels
Flixster	3,000	3,000	26,173	0.5,1,...,5
Douban	3,000	3,000	136,891	1,2,...,5
Yahoo	3,000	3,000	5,335	1,2,...100
ML-100K	943	1,682	100,000	1,2,...,5
ML-1M	6,040	3,706	1,000,209	1,2,...,5
ML-10M	69,878	10,677	10,000,054	0.5,1,...,5

Supplementary Table 1. Statistics of the datasets. The number of users and items indicates the number of rows and columns in the rating matrix, and the number of ratings indicates the observed values in this matrix. The rating levels represent the range of ratings in different datasets.

Supplementary Algorithm Pseudo Code

Algorithm 1 FedPerGNN

Each client constructs its local subgraph $\mathcal{G}_i$
Initialize Θ_i on each user client using the same seed
Iteration count $c \leftarrow 0$
Epoch $e \leftarrow 0$

// Server

repeat
    Select a subset $\mathcal{S}$ from the user set $\mathcal{U}$ randomly
    $\mathbf{g} = 0$
    for each user client $u_i \in \mathcal{S}$ **do**
        if $c|\mathcal{S}| < P$ **then**
            $\mathbf{g} \leftarrow \mathbf{g} + \textbf{LocalGradCal}(\mathcal{G}_i, False)$
        else
            $\mathbf{g} \leftarrow \mathbf{g} + \textbf{LocalGradCal}(\mathcal{G}_i, True)$
        end if
        if $c|\mathcal{S}| \geq e \cdot P$ **then**
            PrivacyPreservingGraphExpansion()
            $e \leftarrow e + 1$
        end if
    end for
    $\mathbf{g} \leftarrow \mathbf{g}/|\mathcal{S}|$
    Distribute **g** to user clients for local update
until model convergence

// User Client

LocalGradCal(i, includeNeighbor):
Select a mini-batch of data $\mathcal{N}$ from $\mathcal{G}_i$
if includeNeighbor **then**
    Use neighboring user embeddings
end if
Compute GNN model gradients $\mathbf{g}_i^m$ and embedding gradients $\mathbf{g}_i^e$ on $\mathcal{N}$
$\mathbf{g}_i \leftarrow (\mathbf{g}_i^m, \mathbf{g}_i^e)$
return $\mathbf{g}_i$

Algorithm 2 Privacy-preserving user-item graph expansion

```
PrivacyPreservingGraphExpansion():
Server sends a public key p to user clients
User clients encrypt item IDs with p
User clients upload the user embedding and encrypted item IDs to a third-party server
Third-party server distributes neighboring user embeddings to user clients
User clients extend local graphs
```
