# Supplementary material for: A federated graph neural network framework for privacy-preserving personalization
Source: Nat Commun. 2022 Jun 2;13:3091. doi: 10.1038/s41467-022-30714-9 (PMC9163103; doi:10.1038/s41467-022-30714-9)
Supplement: Supplementary file 2 — Reporting Summary [file 41467_2022_30714_MOESM2_ESM.pdf]

nature portfolio

Corresponding author(s): Fangzhao Wu, Yongfeng Huang

Last updated by author(s): May 12, 2022

Reporting Summary

Nature Portfolio wishes to improve the reproducibility of the work that we publish. This form provides structure for consistency and transparency in reporting. For further information on Nature Portfolio policies, see our Editorial Policies and the Editorial Policy Checklist.

Statistics

For all statistical analyses, confirm that the following items are present in the figure legend, table legend, main text, or Methods section.

n/a	Confirmed	
☐	☒	The exact sample size (n) for each experimental group/condition, given as a discrete number and unit of measurement
☐	☒	A statement on whether measurements were taken from distinct samples or whether the same sample was measured repeatedly
☐	☒	The statistical test(s) used AND whether they are one- or two-sided<br>*Only common tests should be described solely by name; describe more complex techniques in the Methods section.*
☒	☐	A description of all covariates tested
☐	☒	A description of any assumptions or corrections, such as tests of normality and adjustment for multiple comparisons
☐	☒	A full description of the statistical parameters including central tendency (e.g. means) or other basic estimates (e.g. regression coefficient) AND variation (e.g. standard deviation) or associated estimates of uncertainty (e.g. confidence intervals)
☐	☒	For null hypothesis testing, the test statistic (e.g. F, t, r) with confidence intervals, effect sizes, degrees of freedom and P value noted<br>*Give P values as exact values whenever suitable.*
☒	☐	For Bayesian analysis, information on the choice of priors and Markov chain Monte Carlo settings
☐	☒	For hierarchical and complex designs, identification of the appropriate level for tests and full reporting of outcomes
☒	☐	Estimates of effect sizes (e.g. Cohen's d, Pearson's r), indicating how they were calculated

Our web collection on statistics for biologists contains articles on many of the points above.

Software and code

Policy information about availability of computer code

Data collection	No software was used for data collection.
Data analysis	The FedPerGNN framework and the standard FedAvg algorithm were implemented by the Keras 2.2.4 library with the tensorflow 1.12 backend. The operating system version is Ubuntu 16.04. The Python version is 3.6.9. Analysis was performed by numpy 1.19.5, scipy 1.5.2 and pandas 1.1.5. More details can be found in the manuscript methods section, supplementary materials, and the code repository https://github.com/wuch15/FedPerGNN.

For manuscripts utilizing custom algorithms or software that are central to the research but not yet described in published literature, software must be made available to editors and reviewers. We strongly encourage code deposition in a community repository (e.g. GitHub). See the Nature Portfolio guidelines for submitting code & software for further information.

Data

Policy information about availability of data

All manuscripts must include a data availability statement. This statement should provide the following information, where applicable:

- Accession codes, unique identifiers, or web links for publicly available datasets
- A description of any restrictions on data availability
- For clinical datasets or third party data, please ensure that the statement adheres to our policy

The datasets that support the findings of this study are all publicly available ones, including MovieLens-100K, MovieLens-1M, MovieLens-10M, Flixster, Douban, and YahooMusic. They are widely used benchmarks for testing personalization methods in different scenarios, such as movie and music recommendation. The MovieLens datasets (100K, 1M, and 10M versions) are publicly available at https://grouplens.org/datasets/ movielens/. The Flixster, Douban, and YahooMusic datasets are publicly available at https://github.com/fmonti/mgcnn.

Field-specific reporting

Please select the one below that is the best fit for your research. If you are not sure, read the appropriate sections before making your selection.

- [ ] Life sciences
- [x] Behavioural & social sciences
- [ ] Ecological, evolutionary & environmental sciences

For a reference copy of the document with all sections, see nature.com/documents/nr-reporting-summary-flat.pdf

Behavioural & social sciences study design

All studies must disclose on these points even when the disclosure is negative.

Study description	The study is quantitative experimental based on the real user behaviors on items and their rating feedbacks.
Research sample	The datasets we used are public datasets collected from different websites, including Flixster, Douban, Yahoo Music, and MovieLens.
Sampling strategy	The datasets are predefined and no additional sample size calculation was performed. The sizes of all datasets (from thousands to millions of samples) are large enough to train the machine learning models, as verified by the experiments. The training, validation, and test sets of datasets were randomly selected and there are no overlaps.
Data collection	The datasets are public ones and no data collection process is performed by the authors. The datasets are collected by the original data publisher on the public reviews on the corresponding websites. The Flixster dataset is collected on the Flixster website. The Douban dataset is collected on the Douban website. The Yahoo Music dataset is collected on the Yahoo Music platform. The MovieLens datasets were collected through the MovieLens website. The authors are blind to the data collection process.
Timing	The Flixster dataset is collected from November 2005 to November 2009 (dates are not specified by the original data publisher). The Douban dataset is collected from 2005 (the detailed period is not provided by the original data publisher). The Yahoo Music dataset is collected from 1999 to 2010 (dates and months are not specified by the original data publisher). The MovieLens datasets were collected from September 19th, 1997 to April 22nd, 1998.
Data exclusions	We conduct experiments on the raw collected dataset and do not perform any data exclusion operations.
Non-participation	There is no dropped sample from the original datasets.
Randomization	The datasets were randomly divided into training, validation and test sets. Each sample was assigned to exactly one of these splits. The details are included in the Dataset section in Supplementary Information.

Reporting for specific materials, systems and methods

We require information from authors about some types of materials, experimental systems and methods used in many studies. Here, indicate whether each material, system or method listed is relevant to your study. If you are not sure if a list item applies to your research, read the appropriate section before selecting a response.

Materials & experimental systems

n/a	Involved in the study	
x		Antibodies
x		Eukaryotic cell lines
x		Palaeontology and archaeology
x		Animals and other organisms
x		Human research participants
x		Clinical data
x		Dual use research of concern

Methods

n/a	Involved in the study	
x		ChIP-seq
x		Flow cytometry
x		MRI-based neuroimaging
